# Evaluating Drug-Eluting Balloons Versus Stenting for Provisional Bifurcation Lesions: Short-Term Outcomes and Future Directions

**DOI:** 10.7759/cureus.78436

**Published:** 2025-02-03

**Authors:** Muhammad Ziyad, Syed Tahir Shah, Muhammad Abdur Rauf, Syed Muzammil Shah, Sana Ullah, Rafi Ullah, Muhammad Ilyas Khan

**Affiliations:** 1 Cardiology, Kuwait Teaching Hospital, Peshawar, PAK; 2 Cardiology, Kuwait Teaching Hospital, Peshawar Medical College, Peshawar, PAK

**Keywords:** drug-eluting balloons, major adverse cardiac events, percutaneous coronary intervention, restenosis, stenting, target lesion revascularization

## Abstract

Background

This prospective cohort study aimed to evaluate the short-term clinical outcomes of drug-eluting balloons (DEBs) versus stenting in provisional bifurcation lesions, focusing on procedural success, safety, and major adverse cardiac events (MACE). The study specifically assessed key adverse outcomes, including restenosis, target lesion revascularization (TLR), myocardial infarction (MI), stent thrombosis, and mortality. Bifurcation lesions present significant challenges in interventional cardiology due to their complex anatomy, which contributes to a higher risk of restenosis, side-branch occlusion, and thrombotic events. This study compared the efficacy, safety, and procedural complexity of DEBs and stenting.

Methodology

Fifty patients with bifurcation lesions were enrolled and divided into two equal groups: 25 (50%) treated with DEBs and 25 (50%) treated with provisional stenting. The primary outcome measure was the incidence of MACE, including TLR, MI, stent thrombosis, and death. The study was conducted over one year, with follow-up assessments at one- and three months post-intervention. After the intervention, all patients were put on dual antiplatelet therapy (DAPT) comprising aspirin, clopidogrel, and high-intensity statins. Additionally, other medications were prescribed based on patient-specific needs.

Results

The DEB group exhibited an overall MACE rate of 2 (8%) compared with 3 (12%) in the stenting group (p = 0.58). Restenosis occurred in 1 (4%) of the DEB group and in 2 (8%) of the stenting group, whereas TLR was required in 1 (4%) and 2 (8%) of the patients, respectively. The incidence of MI was low in both groups 1 (4%), with no reported cases of stent thrombosis or death. No significant differences were observed between the groups in any of the clinical outcome measures (p > 0.05). The DEB group achieved treatment success without necessitating side-branch stenting or additional kissing balloon inflation, whereas the stenting group demonstrated comparable success, but increased procedural complexity.

Conclusion

DEBs and stenting showed comparable short-term efficacy and safety for provisional bifurcation lesions, offering the potential advantage of procedural simplicity. This study supports the viability of DEBs as an effective treatment option for selected bifurcation lesions, avoiding the additional complexities associated with stenting. However, larger studies with extended follow-up are necessary to validate these findings and inform clinical decision-making.

## Introduction

Coronary bifurcation lesions remain a challenging subset in interventional cardiology due to their complex anatomy and the risk of restenosis, particularly at the side-branch ostium. While provisional stenting of the main branch, with side-branch intervention only when necessary, has emerged as the preferred approach for most bifurcation lesions, the optimal technique for side-branch treatment remains uncertain [1,2]. Side-branch restenosis can lead to recurrent angina, ischemia, and the need for repeat revascularization, increasing procedural risks and healthcare burden.

Drug-eluting stents (DES) have reduced restenosis rates in the main branch compared to bare-metal stents; however, side-branch ostial restenosis remains a major concern. To address this, various two-stent techniques, including crush, culotte, and T-stenting, have been developed [3]. However, these approaches present specific technical challenges. The crush technique carries a higher risk of stent malapposition, culotte stenting requires multiple rewiring steps, making it more complex, and T-stenting can result in incomplete ostial coverage, increasing the likelihood of restenosis. Studies comparing complex two-stent techniques with provisional stenting have generally shown similar or superior outcomes with the simpler provisional approach, particularly in terms of major adverse cardiac events (MACE), procedural time, and reduced risk of peri-procedural complications [4-6].

Recent research has focused on optimizing the provisional approach through adjunctive techniques, such as proximal optimization (POT) and kissing balloon inflation, which enhances stent apposition and side-branch patency [7]. Additionally, there is growing interest in drug-eluting balloons (DEBs) as an alternative to side-branch stenting in bifurcation lesions. This strategy delivers antiproliferative agents without placing a permanent scaffold, potentially reducing restenosis and preserving future intervention options [8]. Early studies have shown promising results with DCB use in small vessels and in-stent restenosis; however, data on de novo bifurcation lesions are limited [9,10].

Ongoing trials are evaluating the current evidence comparing DCBs with provisional stenting strategies for bifurcation lesions based on short-term angiographic and clinical outcomes. As the complexity of the treated lesions increases, a tailored approach based on individual anatomy may be necessary to achieve optimal results in bifurcation interventions. DEBs versus stenting for provisional bifurcation lesions require a comprehensive understanding of historical developments, key concepts, and clinical challenges [11].

This study aimed to assess the short-term outcomes of these approaches and to explore their potential implications for future practice. In this prospective cohort study, patients with bifurcation lesions treated with DEBs and provisional stenting were evaluated based on the incidence of MACE, including restenosis, target lesion revascularization (TLR), myocardial infarction (MI), stent thrombosis, and death.

## Materials and methods

Study design and duration

The study was designed according to the standard protocols and procedures [12,13]. This study was conducted over one year from July 2023 to June 2024. However, short-term follow-up assessments were conducted at one and three months post-intervention to capture the early incidence of MACE. While the study duration was one year, the primary focus was on short-term outcomes. After the intervention, all patients were put on dual antiplatelet therapy (DAPT) comprising aspirin, clopidogrel, and high-intensity statins. Furthermore, other medications were prescribed depending on the needs of the patient. Follow-up assessments were conducted at one and three months post-percutaneous coronary intervention (PCI) to capture the peak incidence of MACE and assess treatment adherence.

To ensure a comprehensive follow-up, patients were contacted through in-person visits, telephone communication, and electronic message reminders, with remote consultations offered to those experiencing transportation limitations.

Study population and sample size

Fifty participants were divided into 2 equal cohorts: 25 were treated with DEBs and 25 were treated with stents. Using OpenRCT, the sample size was calculated with a 95% confidence level, 80% statistical power, and an anticipated outcome difference of 20% between cohorts.

Inclusion criteria

The study included patients who met the specific inclusion criteria. Participants were required to be at least 18 years old and have a confirmed diagnosis of stable angina or acute coronary syndrome (ACS) necessitating PCI. Moreover, patients were required to present with non-left main distal bifurcation lesions involving the main vessel and one or more side branches, excluding those classified as complex bifurcation lesions based on “Definition Criteria”. Additionally, the lesions were required to be amenable to DEB treatment, wherein side-branch stenting could be avoided or deemed unnecessary.

Exclusion criteria

Patients with specific conditions were excluded from this study. This study included individuals with non-bifurcation or complex bifurcation lesions that necessitated stenting. Patients with severe renal impairment (estimated glomerular filtration rate (eGFR) < 30 mL/min/1.73 m²) were excluded due to the increased risk of contrast-induced nephropathy and poorer post-PCI outcomes, which could confound the evaluation of MACE rates. Furthermore, patients with severe comorbidities, pregnant women, or those with contraindications to antiplatelet therapy were deemed ineligible for participation.

Treatment protocols

In the stenting group, provisional stenting was performed in the main vessel while the side branch remained untreated via percutaneous coronary angioplasty (POBA). To optimize bifurcation, kissing balloon inflation was performed as needed. The decision to perform kissing balloon inflation was made at the operator’s discretion based on anatomical indications.

In the DEB group, neither side branch POBA nor kissing balloon inflation was performed. DEBs were used to deliver antiproliferative agents to the vessel wall to reduce restenosis without the placement of a permanent scaffold.

Data collection procedure

Patient demographics, clinical history, procedural details, and post-PCI outcomes were obtained from hospital records, including both electronic and paper-based systems. Follow-up data at one and three months post-PCI were collected through clinic visits, telephone interviews, and electronic message reminders. Clinical outcomes and adverse events, including therapeutic adherence, were documented in a standardized format.

Outcome measures

In addition to the primary outcomes, this study evaluated several secondary outcomes, including procedural complications such as coronary dissection, no-reflow phenomenon, vascular access complications, and periprocedural myocardial infarction. Short-term adverse events, particularly bleeding incidents, were also assessed. Follow-up data were collected at one and three months post-PCI, as these time points align with the period of peak risk for early MACE and restenosis. While long-term outcomes are clinically relevant, the primary focus of this study was to evaluate early procedural success and safety. Future studies with extended follow-up are warranted to assess long-term efficacy. Bleeding incidents were classified according to the Bleeding Academic Research Consortium (BARC) criteria (grades 2, 3, and 5). Events were adjudicated based on hospital records, clinical examination findings during follow-up visits, and standardized patient-reported outcomes obtained via telephone or electronic consultations. Any ambiguous cases were reviewed by the clinical team to ensure consistency.

Statistical analysis

Statistical analyses were performed using IBM SPSS Statistics for Windows (version 25.0; IBM Corp., Armonk, NY, USA). Continuous variables were presented as mean ± standard deviation (SD) and compared using independent t-tests. Categorical variables are presented as frequencies (n) and percentages (%) and were compared using the chi-square test (χ²). Statistical significance was set at P < 0.05. Key test statistics (e.g., t-value or χ²) were reported, along with p-values, to enhance interpretability.

## Results

In this prospective cohort study, 50 (100%) patients with bifurcation lesions were enrolled and equally assigned to two groups: 25 (50%) treated with DEBs and 25 (50%) treated with provisional stenting. Short-term clinical outcomes were assessed based on the incidence of MACE, including restenosis, TLR, MI, stent thrombosis, and mortality while accounting for various demographic baselines (Table 1).

**Table 1:** Baseline demographics of the study population This table presents the baseline demographics of the two study groups: the DEB group (n = 25) and the stenting group (n = 25). The absence of statistically significant differences between the two groups across all demographic parameters (p > 0.05) suggests that the groups were comparable in terms of baseline characteristics. This comparability ensures an unbiased assessment of outcomes between the two intervention strategies. DEB: drug-eluting balloon

Parameter	DEB Group (n = 25)	Stenting Group (n = 25)	p-value	Test Statistic
Mean age (years)	56.8 ± 9.2	57.4 ± 8.7	0.812	t = 0.24
Male, n (%)	18 (72%)	17 (68%)	0.758	χ² = 0.09
Mean BMI (kg/m²)	27.1 ± 3.6	26.8 ± 3.9	0.771	t = 0.29
Smoking history, n (%)	11 (44%)	12 (48%)	0.781	χ² = 0.07
Diabetes, n (%)	10 (40%)	11 (44%)	0.780	χ² = 0.08
Hypertension, n (%)	12 (48%)	13 (52%)	0.768	χ² = 0.09

As shown in Table 2, the DEB group exhibited an overall MACE incidence of 2 (8%), compared to 3 (12%) in the stenting group (p = 0.58), indicating no statistically significant difference between the two treatment strategies. Restenosis occurred in one (4%) patient in the DEB group and two (8%) in the stenting group. TLR was required in one (4%) of DEB patients, whereas two (8%) in the stenting group underwent TLR. The incidence of MI was low and identical in both groups, with one (4%) in each (p = 1.0). No cases of stent thrombosis or mortality were reported in either group during follow-up.

**Table 2:** Clinical outcomes of DEB vs. stenting groups Short-term clinical outcomes comparing drug-eluting balloons (DEB) and provisional stenting. No significant differences were observed between the groups (p > 0.05) for MACE, restenosis, TLR, MI, stent thrombosis, or mortality. Statistical significance was assessed using chi-square (χ²) tests. MACE: major adverse cardiac events; TLR: target lesion revascularization; MI: myocardial infarction

Outcome	DEB Group (n = 25)	Stenting Group (n = 25)	p-value	Test Statistic
MACE Incidence (n, %)	2 (8%)	3 (12%)	0.580	χ² = 0.31
Restenosis (n, %)	1 (4%)	2 (8%)	0.554	χ² = 0.35
Target Lesion Revascularization (TLR) (n, %)	1 (4%)	2 (8%)	0.554	χ² = 0.35
Myocardial Infarction (MI) (n, %)	1 (4%)	1 (4%)	1.000	χ² = 0.00
Stent Thrombosis (n, %)	0 (0%)	0 (0%)	—	—
Mortality (n, %)	0 (0%)	0 (0%)	—	—
MACE Incidence (n, %)	2 (8%)	3 (12%)	0.580	χ² = 0.31

Overall, no significant differences (p > 0.05) were observed between the DEB and stenting groups for any of the clinical outcome measures. The incidence of MACE, restenosis, TLR, MI, stent thrombosis, and mortality was low and comparable between the two groups, suggesting that both treatment strategies demonstrated similar short-term efficacy and safety in managing bifurcation lesions.

In the DEB group, treatment was successfully completed without the need for side-branch stenting or kissing balloon inflation. The stenting group demonstrated comparable procedural success; however, additional interventions, including side-branch management, increased the procedural complexity.

## Discussion

The findings of this study provide significant insights into the short-term outcomes of DEBs versus stenting for provisional bifurcation lesions. Post-intervention, all patients underwent similar standard medication therapy, consisting of DAPT with aspirin and clopidogrel, along with high-intensity statins. Beyond preventing restenosis, DAPT plays a crucial role in stabilizing vulnerable plaques and reducing thrombotic events while high-intensity statins contribute to plaque stabilization and exert anti-inflammatory effects. These pharmacological benefits likely contributed to the low rates of MACE observed in both groups, reinforcing their comparable safety profiles. These results align with previous research emphasizing the critical role of pharmacological management in PCI. Although the overall incidence of MACE was marginally lower in the DEB group (two (8%) vs. three (12%)), this difference was not statistically significant (p = 0.58). These findings are consistent with existing literature, suggesting that DEBs and stents demonstrate comparable short-term efficacy for certain bifurcation lesions [14]. The observed restenosis and TLR rates were slightly higher in the stenting group, suggesting a potential advantage of DEBs in minimizing restenosis due to the absence of a permanent scaffold. Without a metal scaffold, DEBs reduce the risk of neointimal hyperplasia, particularly benefiting patients with small vessel diameters or those at high risk for restenosis such as individuals with diabetes or diffuse coronary disease. However, the limited sample size and lack of statistical significance (p > 0.05) necessitate a cautious interpretation of these findings. In larger studies, these numerical differences may reach statistical significance, underscoring the need for further research to validate these preliminary observations. The comparable incidence of myocardial infarction (1 (4%)) in both groups and the absence of stent thrombosis or mortality indicate the overall short-term safety of both strategies. The absence of stent thrombosis and mortality may reflect the small sample size and short follow-up period, which limit the ability to detect rare or late-onset events. Extended follow-up is necessary to capture these outcomes, as complications like late stent thrombosis often occur beyond the initial months post-intervention. However, the increased procedural complexity associated with stenting, particularly regarding side branch management, underscores the potential technical advantages of DEBs in select cases. 

The low incidence of major adverse events, including major bleeding, in both groups suggests that both DEB and stenting are safe treatment options for patients with bifurcation lesions. Similarly, the comparable rates of minor bleeding and dyspnea reinforce the equivalent safety profiles of the two interventions. No statistically significant differences were observed between the DEB and stenting groups for any adverse event category (p > 0.05).

Despite these findings, the study's limited sample size and relatively short follow-up period constrain its ability to detect rare events and assess long-term outcomes, such as late restenosis or chronic adverse events. Further studies with larger cohorts and extended follow-up durations are necessary to confirm these results.

Provisional stenting is widely regarded as the gold-standard approach for coronary bifurcation lesions [1,15]. Studies have demonstrated that conservative provisional side-branch intervention strategies yield similar or superior long-term clinical outcomes compared to more aggressive approaches [2,16]. The conservative strategy has been associated with lower rates of procedure-related myocardial necrosis and target vessel failure at three years of follow-up [16]. However, some studies have reported contradictory findings. For instance, Chen et al. (2017) reported that the double-kissing crush stenting technique was associated with lower TLR rates compared to provisional stenting at five years of follow-up, particularly in complex bifurcation lesions [11]. In contrast, Ferenc et al. (2008) found no significant difference in restenosis rates between routine and provisional T-stenting approaches.

While DES are commonly used in bifurcation interventions, the provided literature contains limited data on drug-eluting balloons in this context [5]. Burzotta et al. (2011) compared different types of DES (sirolimus-eluting vs. everolimus-eluting) in bifurcation lesions and found similar procedural performance but improved side-branch outcomes with everolimus-eluting stents. In summary, while provisional stenting has demonstrated favorable outcomes in bifurcation lesions, there remains a lack of direct comparisons with drug-eluting balloons in the existing literature [17].

Ongoing clinical trials and advancements in DEB technology, including enhanced drug delivery profiles and hybrid approaches, may further expand its applicability in bifurcation lesions. Moreover, the integration of intravascular imaging tools, such as optical coherence tomography (OCT), could optimize lesion assessment and procedural planning, potentially leading to improved outcomes.

Study limitations and future directions

A key limitation of this study is its relatively small sample size (50 patients), which may impact the generalizability of the findings. While the results suggest comparable short-term efficacy and safety between DEBs and stenting, larger multicenter studies are required to validate these outcomes in broader patient populations.

## Conclusions

Our findings suggest that drug-eluting balloons (DEBs) demonstrated comparable short-term efficacy and safety to provisional stenting in bifurcation lesions. However, due to the limited sample size and short follow-up period, definitive conclusions regarding non-inferiority cannot be made. The DEB group showed a similar safety profile and efficacy compared to the stenting group, with a non-significant trend toward lower rates of restenosis and target lesion revascularization (TLR).

These findings indicate that DEBs may be a viable treatment option for selected bifurcation lesions, offering the potential benefit of reduced procedural complexity by avoiding permanent scaffold placement and minimizing the need for side-branch interventions. While this study suggests that both DEBs and stenting are effective therapeutic modalities for managing provisional bifurcation lesions, the simplified procedural approach associated with DEBs may make them preferable in certain patient subsets. However, to validate these preliminary findings and assess long-term outcomes, including late restenosis and stent thrombosis, larger multicenter studies with extended follow-up are warranted. Future research should also explore the role of DEBs in more complex bifurcation lesions and identify specific patient populations that may benefit most from this approach, thereby informing clinical decision-making.
